# Einthoven dissertation prizes 2017

**DOI:** 10.1007/s12471-018-1136-y

**Published:** 2018-07-25

**Authors:** J. J. Piek

**Affiliations:** AMC Heart Center, Academic Medical Center, Amsterdam, The Netherlands

The dissertation prize is named after Willem Einthoven, a pioneer in cardiovascular medicine who recorded the first human ECG in 1902, for which he was awarded the Nobel prize in 1924 [1]. The annual Einthoven dissertation prize is an initiative of the Netherlands Heart Institute (NHI) and the Netherlands Society of Cardiology (NVVC) to select the top three cardiovascular theses published in the year 2017. The jury received a total of 26 PhD dissertations for selection. The ranking of the theses was based upon a combination of parameters that included the curriculum vitae of the candidate, the scientific originality of the PhD thesis and its relevance for the cardiovascular field. Moreover, several objective bibliometric parameters were used that included the number of articles in citation index journals, both in PubMed and the Web of Science (WOS), the number of citations in WOS, the Hirsch index and finally the contribution of the candidate as a first author. Based upon this evaluation the jury elected the following nominees: S. Bekkering, N. van Ditzhuijzen, and M. Hazebroek. The members of the jury were P.A. Doevendans and D.J. Duncker (Netherlands Heart Institute), H. Bosker (NVVC), I. van Gelder (CVOI) and M.J. Schalij (President *Concilium Cardiologicum*). The three candidates presented their PhD theses at the NHI meeting in Utrecht on June 28–29^th^ 2018. We congratulate the laureates for their excellent scientific work and their presentations during the meeting.

## Summary

### The dark side of innate immune memory: the development of atherosclerosis

The leading cause of death worldwide is cardiovascular disease due to atherosclerosis. In the Netherlands alone, every day 100 people die from atherosclerosis. Therefore, there is a great need to better understand this disease and its progression. In the last 15 years, the role of inflammation and the immune system in atherosclerosis has been increasingly highlighted. The most important immune cells to play a role in atherosclerosis are monocytes. Monocytes are part of the innate immune system, which is rapid, nonspecific and presumably without any memory. The other part of the immune system is the adaptive immune system, which can acquire a memory by producing memory cells. Recently, however, it was discovered that this dichotomy is not true. Monocytes can develop a memory after a first encounter with a microbial stimulus, after which the cell is functionally reprogrammed. Upon a secondary encounter, the monocyte will exhibit a hyperactive response, this is called ‘trained immunity’. Although this can be of great benefit in the context of recurrent infections, it might be detrimental in conditions in which innate immunity itself contributes to tissue injury. In this thesis, we explored the role of inflammation in the development of atherosclerosis in the light of trained immunity. First, we investigated whether different lipoprotein particles can induce trained immunity, similar to micro-organisms. Second, we examined the possibility of a trained immune phenotype in monocytes from patients with atherosclerosis or patients at risk of developing atherosclerosis. Third, we aimed to unravel part of the mechanisms of trained immunity, which is essential for future drug development.

These questions were explored on several levels: *in vitro, ex vivo* and *in vivo* studying patients with and without atherosclerosis and on a mechanistic level. In the lab, we studied how monocytes respond to a first encounter with different forms of cholesterol: low-density lipoprotein (LDL), lipoprotein(a) (Lp(a)) and oxidised LDL (oxLDL). How do monocytes respond 6 days after a first encounter with these particles, and can they become trained? Firstly, we demonstrated that both oxLDL and Lp(a), two particles carrying oxidised phospholipids, can induce trained immunity *in vitro*, i. e. increased production of atherogenic cytokines and chemokines and increased foam cell formation. Translationally, we demonstrated that monocytes from patients with elevated levels of lipoprotein(a) also show this pro-inflammatory phenotype when stimulated *ex vivo*, indicating a trained immunity phenotype. Secondly, we showed that patients with established atherosclerosis have a different monocyte phenotype in terms of increased cytokine production and changes in the metabolic state compared with healthy controls. Also, patients and controls showed significant differences on an epigenetic level, by decreased histone 3 lysine 27 trimethylation, an inactivating histone mark. Finally, on a mechanistic level, we showed that statins, widely used cholesterol lowering drugs, can prevent the development of trained immunity. We unravelled that the trained immunity phenotype is mediated by accumulation of mevalonate and subsequent stimulation of the insulin-like growth factor (IGF1) receptor. These findings might be used in the future for the development of novel drugs.

*S. Bekkering*

*RadboudUMC, Nijmegen, the Netherlands*

*Siroon.Bekkering@radboudumc.nl*

## Summary

### Coronary artery disease: Assessing the development and treatment of coronary atherosclerosis

Coronary artery disease (CAD) is a major cause of morbidity and mortality. Although animal models increased our understanding of CAD development and treatment, the pathophysiology of CAD, especially in the context of diabetes mellitus (DM), remains incompletely understood. Diabetes mellitus patients have a 2–6-fold higher risk to encounter adverse events associated with CAD than patients without diabetes mellitus, and with the increasing prevalence of diabetes, further studies into CAD development and treatment are mandatory. For this purpose, swine are an excellent model as they can be rendered diabetic, mimicking the human situation of multiple co-morbidities, develop atherosclerotic lesions at anatomical locations similar to humans and allow for coronary stent-implantation and invasive assessment of the coronary vasculature *in vivo*.

This thesis evaluated CAD development and treatment by bioresorbable vascular scaffolds (BVS), while focusing on coronary pathology associated with diabetes mellitus, using intracoronary imaging techniques such as optical coherence tomography (OCT) to obtain highly detailed imaging of the coronary morphology and the vascular response to stent implantation.

First, we used OCT to demonstrate a low incidence of imaging-related complications (0.6%) in an unselected patient population of 1,142 patients with varying indications for imaging. These complications were self-limiting after retrieval of the imaging catheter or easily treatable in the catheterisation laboratory, indicating that OCT is safe to use with a very low event rate. In an experimental setting, we examined the impact of longitudinal catheter displacement on the quantitative assessment of BVS and demonstrated that serial evaluation of matched cross-sections was hampered. This suggested that dedicated analysis methods with per-frame analysis or using software allowing for synchronised evaluation of matched segments, could be more suitable for serial analysis in specific scaffold regions.

Second, OCT and intravascular ultrasound enabled plaque characterisation *in vivo* in diabetic and non-diabetic swine that were fed a high-fat diet and proved to be complementary for the evaluation of CAD development. Using *in vivo* OCT, near-infrared spectroscopy (NIRS) and coronary computed tomography angiography and *ex vivo* vascular function testing and histology, no differences in early atherosclerotic development were observed between diabetic and non-diabetic swine up to 15 months, suggesting that macroscopic atherosclerosis development was not influenced by hyperglycaemia. Additionally, *ex vivo* vascular function testing demonstrated a shift in balance of different contributors to vascular tone of small coronary arteries during the progression of atherosclerosis, emphasising the importance of longitudinal studies of vascular function in diabetes mellitus and CAD.

Third, we used OCT to evaluate the vascular healing response 5 year after first-in-man BVS implantation and demonstrated development of a stable plaque covered by a signal-rich tissue layer, potentially shielding the plaque, in a majority of patients. However, this favourable response was not universal. Additionally, the vascular healing response to BVS implantation was investigated in swine with and without DM using *in vivo* OCT, polarisation-sensitive OCT and NIRS 3 and 6 months after BVS implantation. A highly heterogeneous neointima was observed, suggestive of neoatherosclerosis formation. Importantly, the considerable neoatherosclerosis development under diet-induced dyslipidaemia may point at neoatherosclerosis as an important contributor to BVS failure at long-term.

In conclusion, this thesis demonstrates that 1) OCT is safe to use in clinical practice, 2) dyslipidaemic swine represent an excellent model to evaluate ‘human-like’ CAD development using *in vivo* intracoronary imaging and 3) intracoronary imaging enhances our understanding of the distinct vascular healing patterns after BVS implantation.

*N. van Ditzhuijzen*

*Erasmus MC, Rotterdam, the Netherlands*

*nienkevd@gmail.com*

## Summary

### Unravelling the origins of dilated cardiomyopathy—How genes, viruses and toxic, metabolic, electric and autoimmune disorders interact to cause dilated cardiomyopathy

#### Dilated cardiomyopathy

**Fig. 1:**
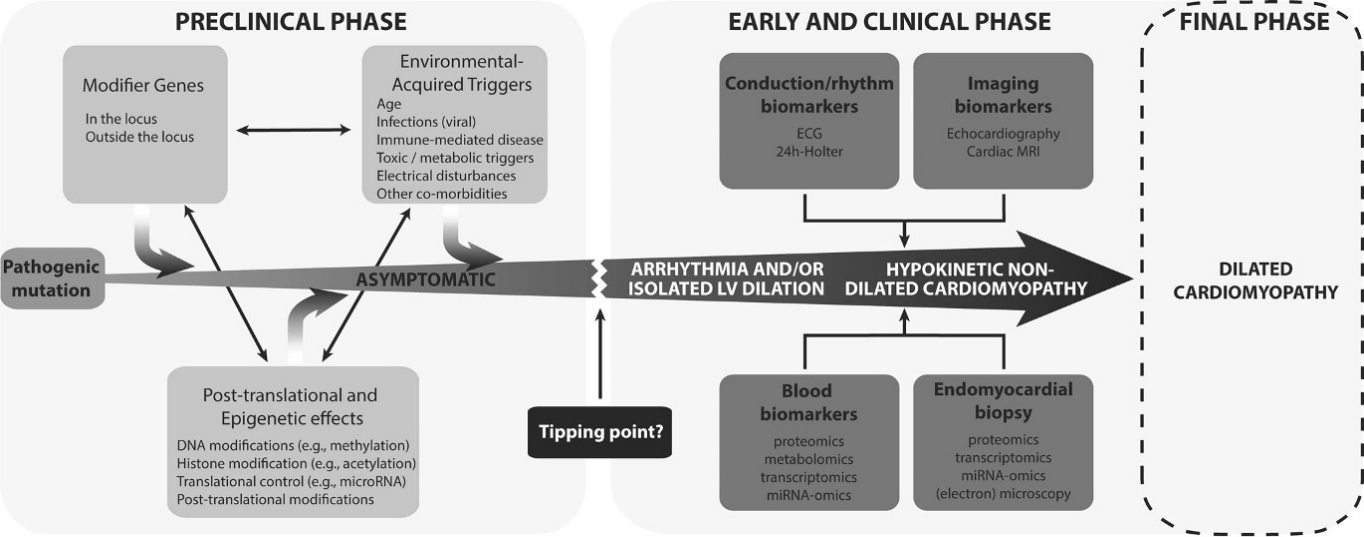
The interplay of genetic and environmental factors reflecting the phenotype and the role of biomarkers during disease progression. (*ECG* electrocardiogram, *MRI* magnetic resonance imaging, *LV* left ventricular)

Heart failure is a complex disease. Even after exclusion of prevalent causes (e. g. hypertension, valvular or ischaemic disease) a multitude of aetiologies may cause heart failure, generally known as ‘idiopathic’ dilated cardiomyopathy (DCM). This disease typically affects younger patients between 20 and 60 years. Possible triggers of DCM are genetic defects, infections (viral), toxic exposure, metabolic abnormalities, electrical disturbances, and immune-mediated diseases [1]. Understanding these underlying causes and pathophysiological pathways are crucial in order to improve aetiology-directed management. Hence, bringing the right treatment to the right person at the right moment. We are at the beginning of unravelling the complexities within this multifactorial disease and standardised aetiology-based treatment strategies are on the horizon. The results in this thesis provide unique insights into novel and known causes, gene-environment interactions, and aetiology-based treatment strategies in DCM patients (Fig. 1).

#### Potential triggers of DCM

Our aim was to explore known and novel triggers of DCM. We demonstrate that the prevalence of gene mutations in a precursor of DCM, recently defined as hypokinetic non-dilated cardiomyopathy [2], is similar to that in DCM. Together with the unfavourable impact on outcome of genetic mutations, irrespective of phenotype, these results provide novel insights into the underlying genetic background and its prognosis in early DCM [3]. In the future, identifying mutation carriers (i. e. patients at risk of developing DCM) in an early stage may lead to improved management and treatment. Moreover, we recommend cardiac screening in autoimmune diseases, particularly in ANCA vasculitis, as cardiac involvement is seen in ~30–50% and predicts mortality [4]. To discover novel causes, we performed an extensive metabolic screening which revealed propionic acidaemia as a novel cause for adult-onset DCM, to date only described in newborns and childhood [5]. Finally, we provide a comprehensive overview of the most frequently found cardiotropic virus, parvovirus B19, in DCM and myocarditis patients and attempt to translate the lessons learned into opportunities for future studies and possible treatment strategies [6].

#### Gene-environment interactions

As mentioned before, DCM is a heterogeneous and complex disease. So far, studies evaluated either genetic or environmental-acquired aetiologies of DCM, with limited knowledge regarding prognosis. The recent MOGE(S) classification system for cardiomyopathies (Morphology, Organ Involvement, Genetic or familial, Etiology, Stage of disease) provides a solution by combining phenotype, genetic variation and etiological annotation [7]. Using extensive genotyping and phenotyping, including endomyocardial biopsies and genetic evaluation, we show that this MOGE(S) classification is applicable and has prognostic value in a large cohort of DCM patients. Importantly, the presence of multiple aetiologies was a strong predictor of outcome [8]. These unique results support the hypothesis that exposure to an environmental and/or acquired trigger in addition to an ‘underlying genetic background’ can have detrimental effects on the prognosis of a patient, as shown again later by our group [9].

#### Aetiology-based treatment strategies

Aetiology-based, personalised therapy is the future. Our results demonstrate the beneficial effects of immunosuppressive therapy on both short-term and long-term outcomes in patients with virus-negative inflammatory cardiomyopathy [10]. This further supports the beneficial effects from previous studies on short-term outcome, and provides new favourable evidence for long-term outcome as well.

*M. Hazebroek*

*Department of Cardiology, Maastricht University Medical Centre* *+, Maastricht, the Netherlands*

*mark.hazebroek@mumc.nl*

Siroon Bekkering won the first prize, Mark Hazebroek the second prize and Nienke van Ditzhuijzen the third prize.
